# The Arnold Berliner Award 2021 honors research on bombardier beetles

**DOI:** 10.1007/s00114-021-01765-7

**Published:** 2021-10-12

**Authors:** Matthias Waltert

**Affiliations:** University of Göttingen, Göttingen, Germany

This is to announce the winner of the 2021 Arnold Berliner Award. *The Science of Nature* grants the Arnold Berliner Award to the lead authors of articles distinguished by their excellent, original, and—especially—interdisciplinary research. As such, the awarded articles ideally reflect the vision of Arnold Berliner (Autrum 1988; Thatje 2018). The award is sponsored by Springer Nature and includes the Arnold Berliner Award medal (Fig. 1), a biennial subscription to the journal’s electronic edition, a 500-Euro voucher for Springer ebooks, and a cash prize of 250 Euro. I am very proud that, this year, the board of editors has decided to award Athula B. Attygalle (Fig. 2) for his article “Biosynthetic origin of benzoquinones in the explosive discharge of the bombardier beetle *Brachinus elongatulus*”. Together with co-authors Sihang Xu, Wendy Moore, Reilly McManus, Aman Gill, and Kipling Will, he described the mechanisms behind one of the most amazing defense mechanisms in animals: the explosive discharge of bombardier beetles. They identified the molecules and pathways that lead to the production of two different benzoquinones. In a stress situation, beetles mix hydrogen peroxide and hydroquinones, which are stored in separate reservoirs, to generate an explosion at high temperatures. Researchers developed innovative methodology to examine the reservoir chambers of the beetles to explore the biochemistry of this defense mechanism and demonstrated that one of the beetle’s benzoquinones is derived from hydroquinone, while the other originated from a completely separate precursor: m-cresol. In particular, authors revisited the chemistry of benzoquinone production by insects to find that not one, but two independent pathways evolved to generate the different benzoquinone forms that generate the explosion when mixed. The study provides much new insight into an absolutely classic and compelling system, with the potential of broader implications for understanding the evolution of insect chemical defenses. On behalf of the board of editors, I congratulate Athula B. Attygalle on the award.

**Fig. 1:**
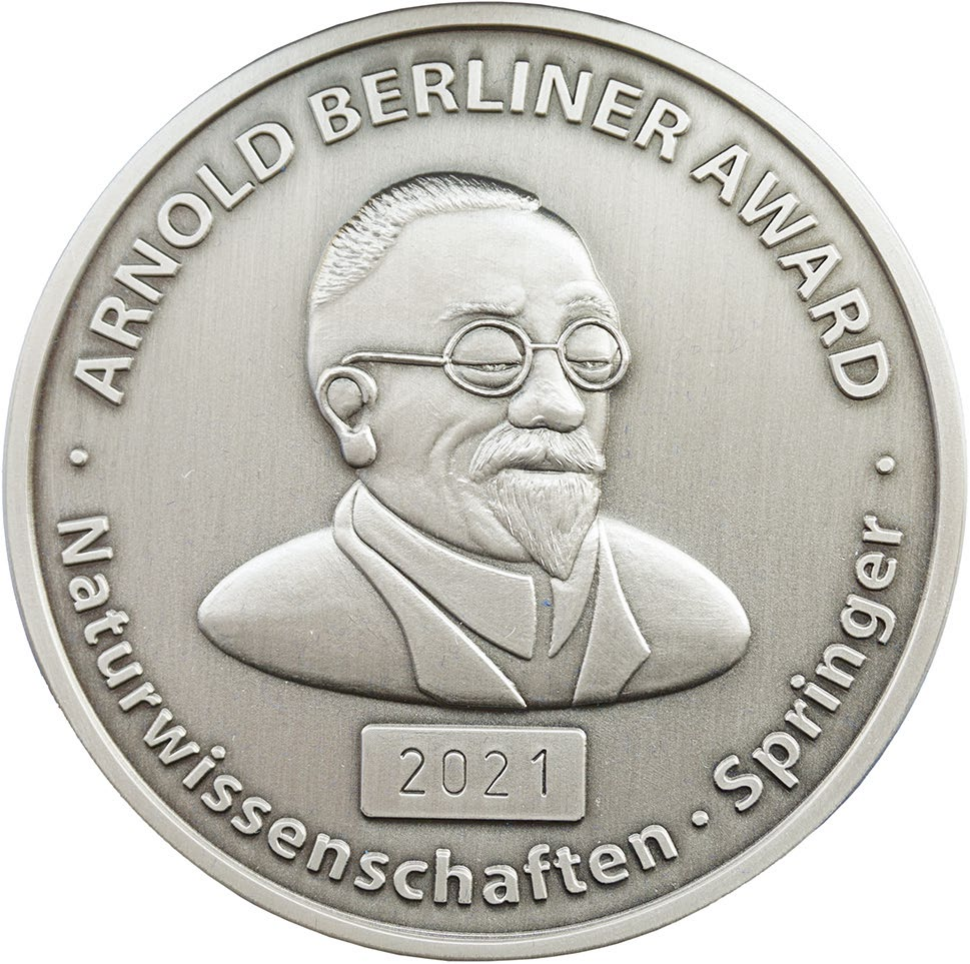
The Arnold Berliner Award medal

**Fig. 2:**
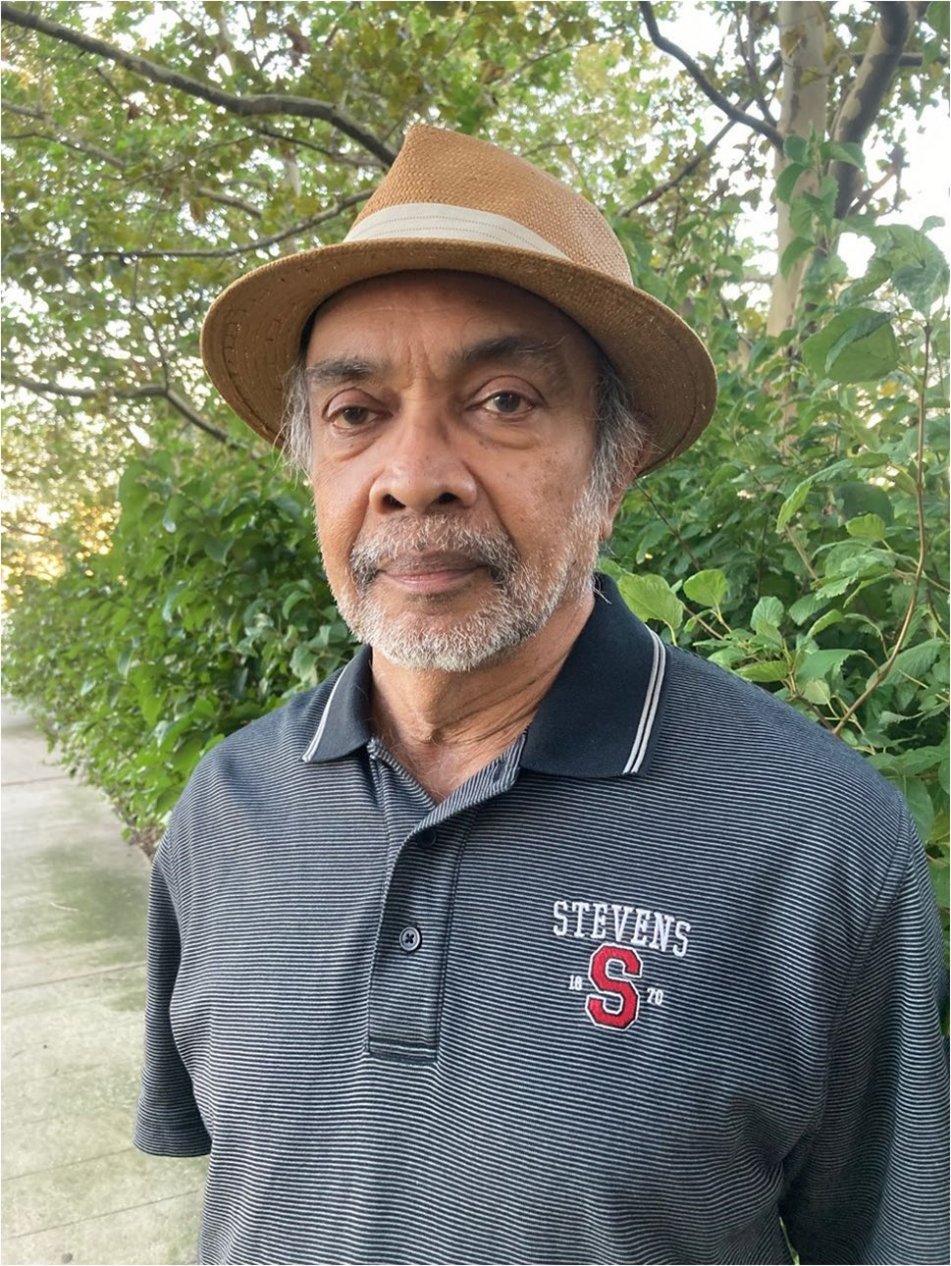
Professor Athula Attygalle

## References

[CR1] Attygalle AB, Xu S, Moore W, McManus R, Gill A, Will K (2020). Biosynthetic origin of benzoquinones in the explosive discharge of the bombardier beetle Brachinus elongatulus. Sci Nat.

[CR2] Autrum H (1988). Arnold Berliner und die“Naturwissenschaften”. Naturwissenschaften.

[CR3] Thatje S (2018). The Arnold Berliner Award 2018. Sci Nat.

